# A Pre-Existing Myogenic Temporomandibular Disorder Increases Trigeminal Calcitonin Gene-Related Peptide and Enhances Nitroglycerin-Induced Hypersensitivity in Mice

**DOI:** 10.3390/ijms21114049

**Published:** 2020-06-05

**Authors:** Hui Shu, Sufang Liu, Yuanyuan Tang, Brian L. Schmidt, John C. Dolan, Larry L. Bellinger, Phillip R. Kramer, Steven D. Bender, Feng Tao

**Affiliations:** 1 Department of Biomedical Sciences, Texas A&M University College of Dentistry, Dallas, TX 75246, USA; hshu@exchange.tamu.edu (H.S.); sufang.liu@tamu.edu (S.L.); yuanyuan8402@163.com (Y.T.); larry-l-bellinger@tamu.edu (L.L.B.); pkramer@tamu.edu (P.R.K.); 2 Department of Physiology and Neurobiology, Zhengzhou University School of Medicine, Zhengzhou 450001, Henan, China; 3 School of Basic Medical Sciences, Xinxiang Medical University, Xinxiang 453003, Henan, China; 4 Bluestone Center for Clinical Research, New York University, New York, NY 10003, USA; brianl.schmidt@nyu.edu (B.L.S.); john.dolan@nyu.edu (J.C.D.); 5 Center for Facial Pain and Sleep Medicine, Texas A&M University College of Dentistry, Dallas, TX 75246, USA; benderdds@tamu.edu; 6 Center for Craniofacial Research and Diagnosis, Texas A&M University College of Dentistry, Dallas, TX 75246, USA

**Keywords:** temporomandibular disorder, migraine pain, comorbidity, spinal trigeminal nucleus caudalis, calcitonin gene-related peptide

## Abstract

Migraine is commonly reported among patients with temporomandibular disorders (TMDs), especially myogenic TMD. The pathophysiologic mechanisms related to the comorbidity of the two conditions remain elusive. In the present study, we combined masseter muscle tendon ligation (MMTL)-produced myogenic TMD with systemic injection of nitroglycerin (NTG)-induced migraine-like hypersensitivity in mice. Facial mechanical allodynia, functional allodynia, and light-aversive behavior were evaluated. Sumatriptan, an FDA-approved medication for migraine, was used to validate migraine-like hypersensitivity. Additionally, we examined the protein level of calcitonin gene-related peptide (CGRP) in the spinal trigeminal nucleus caudalis using immunohistochemistry. We observed that mice with MMTL pretreatment have a prolonged NTG-induced migraine-like hypersensitivity, and MMTL also enabled a non-sensitizing dose of NTG to trigger migraine-like hypersensitivity. Systemic injection of sumatriptan inhibited the MMTL-enhanced migraine-like hypersensitivity. MMTL pretreatment significantly upregulated the protein level of CGRP in the spinal trigeminal nucleus caudalis after NTG injection. Our results indicate that a pre-existing myogenic TMD can upregulate NTG-induced trigeminal CGRP and enhance migraine-like hypersensitivity.

## 1. Introduction

Both temporomandibular disorder (TMD) and migraine are highly prevalent and debilitating conditions [1,2]. TMD is a set of complex conditions of the masticatory muscles, the temporomandibular joint (TMJ), and associated structures. The prevalence of TMD has been reported as 5% in the United States according to the Orofacial Pain Prospective Evaluation and Risk Assessment (OPPERA) study [3] and between 5% and 12% worldwide [4]. Among all the signs and symptoms produced by TMD, pain is the most significant as it directly reduces quality of life and the daily activities of affected individuals [5]. The etiology of TMD pain is multifactorial, including arthrogenic- and myogenic-originated disorders. On the other hand, migraine is the most disabling of all neurological disorders [6,7]. Migraine headache is a common primary headache. The mechanisms underlying migraine headache have been studied for many years, yet it remains unclear how transition from acute to chronic migraine headache occurs and whether other facial pain conditions (e.g., TMD) affect chronicity of migraine headache.

Growing epidemiological data suggest that TMD and migraine are closely associated [8,9]. It has been reported that the presence of migraine contributes to the risk of developing TMD [10]. Meanwhile, migraine and other headaches appear to be more prevalent in TMD population [11], especially in the individuals with myogenic TMD [12]. A clinical study indicates that patients with a comorbidity of TMD and migraine have a more severe condition [13]. TMD and migraine may share common pathophysiological aspects, and simultaneous treatment approaches to the two diseases could be more effective than separate therapies [14]. However, the comorbidity of migraine and TMD remain poorly understood.

Previous studies have shown that masseter muscle tendon ligation (MMTL) in rats can produce myogenic TMD pain [15,16] and that systemic injection of nitroglycerin (NTG) can induce acute migraine-like hypersensitivity [17,18,19,20]. An essential role for calcitonin gene-related peptide (CGRP) has been indicated in migraine pathophysiology [21,22,23]. In the present study, by combining the MMTL-produced myogenic TMD with NTG-induced migraine-like hypersensitivity, we investigated the effect of a pre-existing myogenic TMD on migraine-like pain and the protein level of CGRP in the spinal trigeminal nucleus caudalis (Sp5C).

## 2. Results

### 2.1. MMTL Produces a Myogenic TMD in Mice 

**Figure 1:**
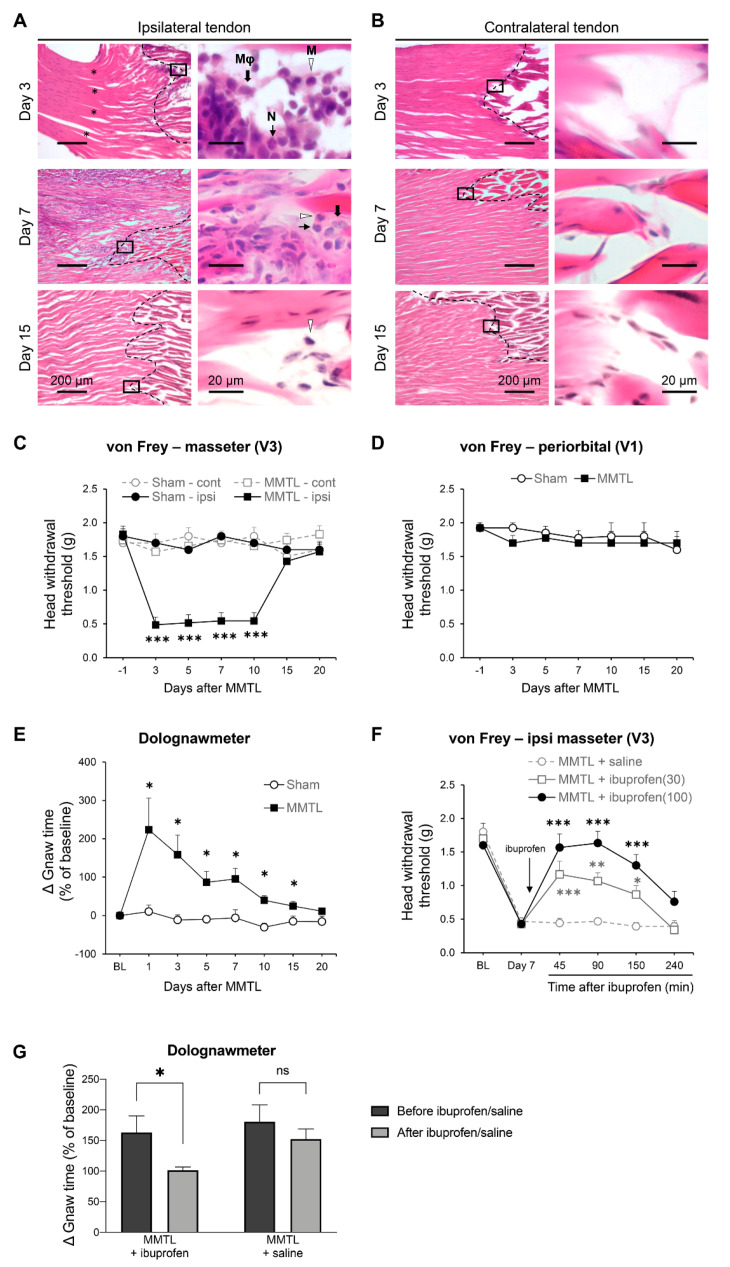
MMTL produces inflammation in the tendon tissue, masseter muscle allodynia, and gnawing dysfunction. (**A**,**B**) Representative H&E staining images of ipsilateral (**A**) and contralateral (**B**) masseter tendon on days 3, 7, and 15 after MMTL. The right panels of the images display the respective boxed areas in the left panels at higher magnification. The asterisks (*) indicate the site of the MMTL injury. The dashed lines indicate the edge between tenocytes and masseter muscle cells. Mϕ, macrophage; M, monocyte; N, neutrophil. The representative staining was repeated three times to confirm our results. (**C**,**D**) MMTL decreased the head withdrawal threshold in the skin area over the masseter muscle (*n* = 6–7; (**C**)), but not in the periorbital region ipsilateral to MMTL surgery (*n* = 6–7; (**D**)). F_treatment_ (3, 22) = 48.990, *p* < 0.001. F_time_ (6, 132) = 7.637, *p* < 0.001; F_interaction_ (18, 132) = 8.466, *p* < 0.001 for (**C**). F_treatment_ (1, 11) = 6.076, *p* = 0.031. F_time_ (6, 66) = 0.860, *p* = 0.529; F_interaction_ (6, 66) = 0.819, *p* = 0.560 for (**D**). (**E**) MMTL increased gnaw time (sham, *n* = 8; MMTL, *n* = 10). F_treatment_ (1, 16) = 10.750, *p* < 0.05; F_time_ (7, 112) = 5.261, *p* < 0.001; F_interaction_ (7, 112) = 3.780, *p* < 0.001. *** *p* < 0.001, ** *p <* 0.01, * *p <* 0.05 vs. the respective sham or saline control. (**F**) Oral administration of ibuprofen produced a dose-dependent increase in head-withdrawal threshold in the skin area over the masseter muscle in mice with MMTL (*n* = 6 per group). F_treatment_ (2, 15) = 17.260, *p* < 0.001; F_time_ (5, 75) = 46.370, *p* < 0.001; F_interaction_ (10, 75) = 6.906, *p* < 0.001. *** *p* < 0.001, ** *p <* 0.01, * *p <* 0.05 vs. the respective sham or saline control. (**G**) Oral administration of ibuprofen (100 mg/kg) on day 7 post MMTL significantly decreased the gnaw time in the dolognawmeter assay, while saline treatment did not have such effect (*n* = 6 per group). * *p* < 0.05 vs. before treatment by Student’s t-test. ns: no significance; MMTL: masseter muscle tendon ligation; ipsi: ipsilateral; cont: contralateral; BL: baseline; ibuprofen(30): 30 mg/kg of ibuprofen; ibuprofen(100): 100 mg/kg of ibuprofen.

Ligation of the anterior superficial part of the masseter muscle tendon (MMTL) has been developed and well-characterized as a long lasting myogenic orofacial pain model in rats [15,16]. In this study, we used the MMTL with smaller ligatures to produce a myogenic TMD in mice. Using H&E staining, we observed that the MMTL caused depressed collagen fiber bundles on the ipsilateral side and inflammatory cells (macrophage, monocyte, and neutrophil) clustered adjacent to the ligated tendon on day 3 after ligation. The inflammatory cell infiltration continued to day 7 and almost disappeared on day 15 post-MMTL (Figure 1A). The contralateral tissue had no inflammatory cell infiltration throughout the time course (Figure 1B). 

To examine whether MMTL causes TMD-like orofacial pain, we used von Frey filaments to measure head withdrawal responses to mechanical stimuli over the masseter muscle (innervated by trigeminal nerve V3 branch) as well as periorbital region (innervated by trigeminal nerve V1 branch) (Figure 1C,D).

Our results showed that the MMTL decreased the head withdrawal threshold responding to ipsilateral stimulation over the masseter muscle for at least 10 days compared with the sham control group, and that the ligation surgery had no effect on the head withdrawal threshold on the contralateral side (Figure 1C).

We also observed that the MMTL did not affect the head withdrawal threshold in the periorbital area throughout the entire time course (Figure 1D).

Furthermore, using a dolognawmeter, a validated operant assay for functional allodynia test, we found that the MMTL significantly increased gnaw time for 15 days compared with the sham control group (Figure 1E). These results indicate that the MMTL causes ipsilateral orofacial pain in the facial areas innervated by trigeminal nerve V3, but not V1, and induces functional allodynia-driven oral dysfunction.

To further validate the MMTL-produced myogenic TMD model, we performed oral gavage of ibuprofen, a first-line and commonly used nonsteroidal anti-inflammatory drug (NSAID) for the treatment of inflammatory TMD [24,25,26,27], on day 7 after MMTL, and we observed that oral administration of ibuprofen dose-dependently inhibited MMTL-caused myofascial pain in the trigeminal nerve V3-innervated masseter area (Figure 1F) and significantly diminished functional allodynia (Figure 1G).

### 2.2. MMTL Enhances NTG-Induced Migraine-Like Hypersensitivity 

**Figure 2:**
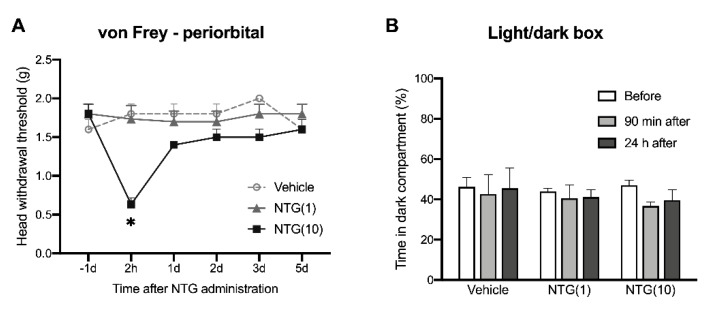
Systemic injection of NTG induces acute migraine-like hypersensitivity in mice. (**A**) Intraperitoneal injection (i.p.) of NTG at 10 mg/kg decreased the head withdrawal threshold in the periorbital region, whereas 1 mg/kg of NTG had no effect on the threshold (*n* = 6 per group). F_treatment_ (2, 14) = 6.394, *p* < 0.05; F_time_ (5, 70) = 2.886, *p* < 0.05; F_interaction_ (10, 70) = 4.189, *p* < 0.001. * *p* < 0.05 vs. the corresponding time point in vehicle group. (**B**) Both vehicle and NTG did not significantly alter the time spent in the dark compartment (*n* = 6–9 per group). NTG: nitroglycerin; NTG(1): 1 mg/kg of nitroglycerin; NTG(10): 10 mg/kg of nitroglycerin.

We injected (i.p.) two different doses of NTG (10 mg/kg and 1 mg/kg) into mice and compared their effects. We observed that the higher dose (10 mg/kg) of NTG markedly decreased the head withdrawal threshold in the periorbital area (Figure 2A) and masseter area (Supplementary Figure S1) at 2 h post-injection, but did not induce light-aversive behavior at 90 min or 24 h after NTG injection (Figure 2B). However, the injection with the lower dose (1 mg/kg) of NTG had no effect on the head withdrawal threshold in the periorbital or masseter area and the light-aversive behavior, compared to the vehicle-treated group (Figure 2, Supplementary Figure S1).

**Figure 3:**
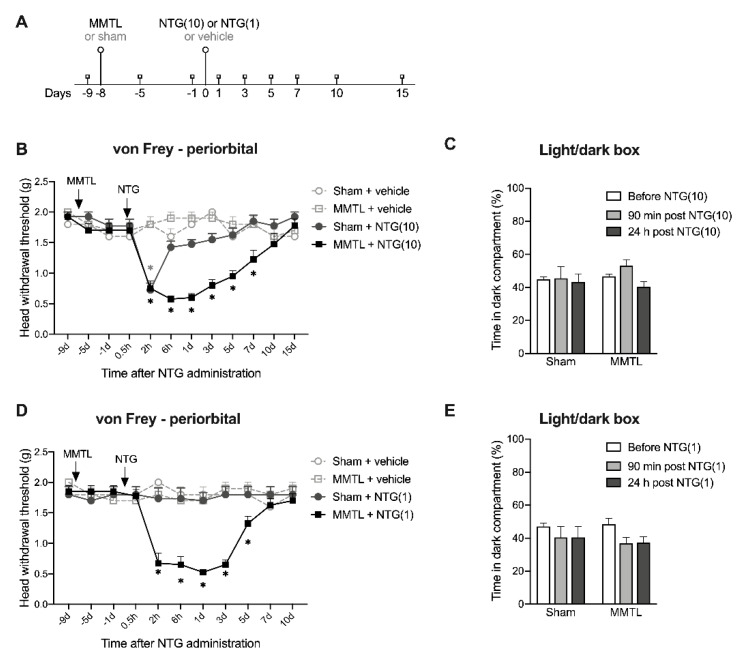
MMTL enhances NTG-induced migraine-like hypersensitivity. (**A**) Timeline of this experiment. (**B**) MMTL pretreatment prolonged the higher dose (10 mg/kg) of NTG-decreased head withdrawal threshold in the periorbital region (*n* = 6–8 per group). F_treatment_ (3, 24) = 61.580, *p* < 0.001. F_time_ (11, 264) = 10.990, *p* < 0.001; F_interaction_ (33, 264) = 7.143, *p* < 0.001. (**C**) NTG (10 mg/kg) with or without MMTL pretreatment did not induce light-aversive behavior at 90 min or 24 h after NTG (*n* = 5–9 per group). (**D**,**E**) MMTL pretreatment enabled the lower dose (1 mg/kg) of NTG to decrease the head withdrawal threshold in the periorbital region (*n* = 6–8 per group; **D**), but did not induce the light-aversive behavior at 90 min or 24 h after NTG (*n* = 5–8 per group; **E**). F_treatment_ (3, 22) = 22.660, *p* < 0.001; F_time_ (10, 220) = 6.787, *p* < 0.001; F_interaction_ (30, 220) = 7.433, *p* < 0.001 for (**D**). *** *p* < 0.001, ** *p* < 0.01, * *p* < 0.05 vs. the corresponding time points in the “sham + vehicle” group (**B**,**D**). MMTL: masseter muscle tendon ligation; NTG: nitroglycerin; NTG(1): 1 mg/kg of nitroglycerin; NTG(10): 10 mg/kg of nitroglycerin.

To investigate whether a pre-existing myogenic TMD affects NTG-induced migraine-like hypersensitivity, we performed MMTL eight days prior to NTG injection (Figure 3A).

We observed that the MMTL surgery (“MMTL + vehicle” group) did not affect the head withdrawal threshold in the periorbital area, while the MMTL pretreatment prolonged the higher dose (10 mg/kg) of NTG-induced acute allodynia in the periorbital area. Notably, NTG combined with the MMTL pretreatment (“MMTL + NTG(10)” group) produced long-lasting mechanical allodynia in the ipsilateral periorbital area for at least seven days (Figure 3B), but only slightly prolonged NTG-induced migraine-like hypersensitivity on the contralateral side (Supplementary Figure S2), whereas NTG without the MMTL pretreatment (“Sham + NTG(10)” group) only significantly decreased the head withdrawal threshold in the periorbital area at 2 h post-injection.

We also observed that NTG (10 mg/kg) with or without MMTL pretreatment did not induced light-aversive behavior at 90 min or 24 h following NTG administration (Figure 3C).

Moreover, we found that the MMTL pretreatment enabled the lower dose (1 mg/kg) of NTG to induce mechanical allodynia in the periorbital area, though the lower dose of NTG alone had no effect on the head withdrawal threshold in the periorbital area (Figure 2 and Figure 3D). The MMTL pretreatment-enabled migraine-like hypersensitivity persisted for five days after the injection with the lower dose of NTG (Figure 3D), but the MMTL pretreatment did not induce light-aversive behavior at 90 min or 24 h after NTG (Figure 3E). 

### 2.3. Sumatriptan Inhibits MMTL-Enhanced Migraine-Like Hypersensitivity 

**Figure 4:**
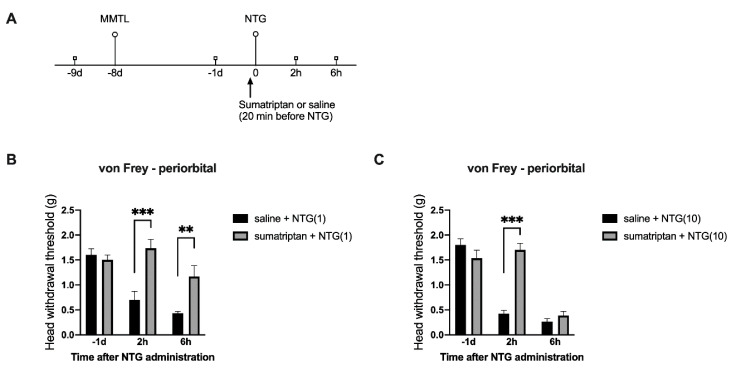
Sumatriptan inhibits MMTL-enhanced migraine-like hypersensitivity. (**A**) Timeline of this experiment. (**B**) On day 8 after MMTL, systemic injection of sumatriptan (i.p., 1 mg/kg) significantly inhibited MMTL-enabled migraine-like hypersensitivity following the injection of the lower dose (1 mg/kg) of NTG at both 2 h and 6 h post-NTG (*n* = 6 per group). *** *p <* 0.001, ** *p <* 0.01 vs. the corresponding time points in the control group (“saline + NTG(1)”). F_treatment_ (1, 10) = 13.950, *p* = 0.004; F_time_ (2, 20) =16.220, *p* < 0.01; F_interaction_ (2, 20) = 9.904, *p* = 0.001. (**C**) On day 8 after MMTL, systemic injection of sumatriptan (i.p., 1 mg/kg) significantly inhibited MMTL-enhanced migraine-like hypersensitivity following the injection of the higher dose (10 mg/kg) of NTG at 2 h post-NTG (*n* = 6 per group). *** *p <* 0.001 vs. the corresponding time points in the control group (“saline + NTG(10)”). F_treatment_ (1, 10) = 20.140, *p* = 0.001; F_time_ (2, 20) = 67.500, *p* < 0.001; F_interaction_ (2, 20) = 24.000, *p* < 0.001. MMTL: masseter muscle tendon ligation; NTG: nitroglycerin; NTG(1): 1 mg/kg of NTG; NTG(10): 10 mg/kg of NTG; -1d: one day before NTG.

To validate that the MMTL pretreatment-enhanced periorbital hypersensitivity after NTG administration is migraine-like, we injected (i.p.) sumatriptan, an FDA-approved drug prescribed for migraine, into mice prior to NTG administration (Figure 4A). We found that compared with the saline control group, sumatriptan significantly inhibited the periorbital hypersensitivity at 2 h and 6 h post-NTG in the lower dose (1 mg/kg) of NTG group (Figure 4B) as well as 2 h post-NTG in the higher dose (10 mg/kg) of NTG group (Figure 4C). 

### 2.4. MMTL Upregulates NTG-Induced CGRP in the Sp5C

**Figure 5:**
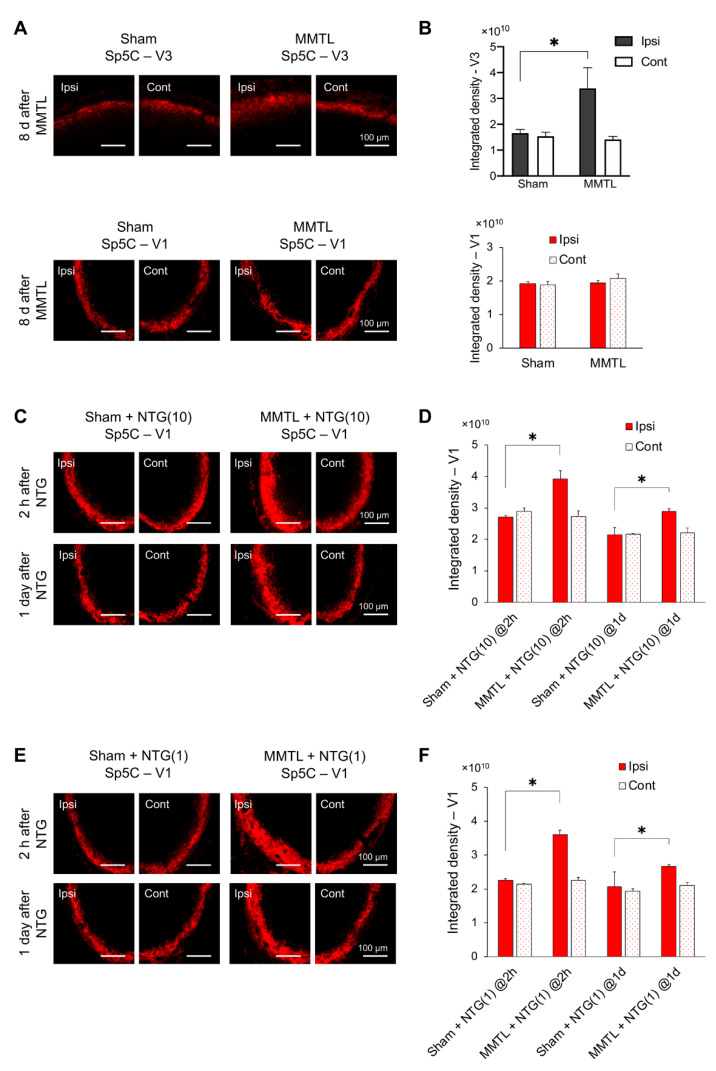
Effect of MMTL pretreatment on the protein level of CGRP in the Sp5C after NTG injection. (**A**) Representative immunofluorescent staining images of CGRP in the rostral Sp5C-V3 and caudal V1 area from sham and MMTL groups on day 8 after surgery. (**B**) Quantification of integrated densities of CGRP in the Sp5C (*n* = 3 mice per group). (**C**,**E**) Representative immunofluorescent staining images of CGRP in the caudal Sp5C-V1 from sham or MMTL plus the higher dose (10 mg/kg) of NTG groups (**C**), and sham or MMTL plus the lower dose (1 mg/kg) of NTG groups (**E**). (**D**,**F**) Quantification of integrated densities of CGRP in the caudal Sp5C-V1 (*n* = 3 mice per group). Note that MMTL significantly increased CGRP in the ipsilateral rostral Sp5C-V3, but had no effect on the CGRP protein level in the caudal Sp5C-V1 compared with the sham-treated group (**A**,**B**), and that MMTL pretreatment significantly increased CGRP in the ipsilateral caudal Sp5C-V1 after NTG injection of both higher dose (10 mg/kg, **E**, **F**) and lower dose (1 mg/kg, **C** and **D**). * *p <* 0.05 vs. the respective control groups. F_treatment_ (1, 8) = 3.725, *p* = 0.90; F_side_ (1, 8) = 6.384, *p <* 0.05; F_interaction_ (1, 8) = 4.951, *p* = 0.057 for the rostral Sp5C-V3. F_treatment_ (1,8) = 1.472, *p* = 0.260; F_side_ (1,8) = 0.225, *p* = 0.648; F_interaction_ (1,8) = 0.838, *p* = 0.387 for the caudal Sp5C-V1 in (**B**). F_treatment_ (3,16) = 15.100, *p* < 0.001; F_side_ (1,16) = 11.28, *p* < 0.005; F_interaction_ (3,16) = 6.426, *p* < 0.05 for (**D**). F_treatment_ (3,16) = 10.750, *p* < 0.001; F_side_ (1,16) = 20.07, *p* < 0.005; F_interaction_ (3,16) = 5.746, *p* < 0.05 for (**F**). CGRP: calcitonin gene-related peptide; MMTL: masseter muscle tendon ligation; NTG: nitroglycerin; NTG(1): 1 mg/kg of nitroglycerin; NTG(10): 10 mg/kg of nitroglycerin.

To investigate the possible mechanism underlying the effect of MMTL on NTG-induced migraine-like hypersensitivity, we examined the protein level of CGRP, a neuropeptide that plays an important role in migraine [28], in the Sp5C. Compared to the sham-operated group, MMTL surgery increased CGRP in the ipsilateral rostral Sp5C (V3), but did not alter the protein level of CGRP in the caudal Sp5C (V1) on day 8 post-MMTL (Figure 5A,B). We further observed that MMTL pretreatment significantly increased CGRP in the ipsilateral Sp5C-V1 after NTG injection at both 2 h and 1 day post-NTG in the higher dose (10 mg/kg) of NTG group (Figure 5C,D) and in the lower dose (1 mg/kg) of NTG group (Figure 5E,F). 

## 3. Discussion

One of the common experiences reported among individuals with TMD is the regular occurrence of headaches [29,30]. It has been proposed that TMD may create a pro-nociceptive environment predisposing the patients to headaches [31]. Currently there is no animal model available for studying the comorbidity of migraine and TMD. Establishing an animal model that mimics the occurrence of enhanced migraine-like hypersensitivity in TMD patients is the central premise for investigating such comorbid condition. In the present study, we combined a myogenic TMD with NTG-induced migraine-like hypersensitivity to develop a comorbidity mouse model, in which we demonstrate for the first time that a pre-existing myogenic TMD enhances NTG-induced migraine-like hypersensitivity in mice. Given that female sex hormones (such as estrogen) fluctuation during menstrual cycle may affect the comorbidity, we used male mice in the present study. Recently, we further observed that the myogenic TMD-enhanced migraine-like hypersensitivity in female mice lasts slightly longer than that in male mice (Supplementary Figure S3). In the future, we will investigate potential sex difference in the comorbidity of TMD and migraine-like pain.

Several mouse models have been used to study TMD, including genetic mouse models [32,33], partial discectomy-induced osteoarthritis in TMJ [34], and TMD induced by algetic agents [35,36,37,38]. These TMD models have helped understand mechanisms related to TMD pain. In this study, we modified a MMTL-based myogenic orofacial pain rat model [15] into a myogenic TMD model in mice. In this myogenic TMD mouse model, long-lasting mechanical allodynia indicates that MMTL can be used to induce TMD pain in mice. Previous studies have reported that clinical TMD pain is associated with oral dysfunction [39,40]. To assess whether the TMD pain in our MMTL mouse model is accompanied with oral dysfunction, we used a dolognawmeter, a validated operant assay [41], to examine mouse gnawing function. Gnaw time in the mice significantly increased for fifteen days post-MMTL, indicating the presence of functional allodynia after the MMTL. Moreover, H&E staining showed that inflammatory cells cluster around the injured tendon at the early stage and then gradually infiltrate into the tendon. The time course of the MMTL-produced local inflammation is consistent with that of the MMTL-induced facial mechanical allodynia, suggesting that there is a correlation between local inflammation and TMD pain following MMTL. Furthermore, using ibuprofen, a first-line and commonly used NSAID for the treatment of inflammatory TMD [24,25,26,27], we validate the MMTL-produced myogenic TMD model. Given that the surrounding structures, such as paratenon and endotenon, are innervated by nerve fibers and the innervation could be damaged by tendon ligation and that there is potential nerve ingrowth into the tendon during tendon repair [42], neuropathic factors may be also involved in the MMTL-produced myogenic TMD. 

Many patients with TMD report pain comorbidities including migraine headache [12]. In this study, we focused on the comorbid myogenic TMD and migraine-like hypersensitivity. A systemic injection of NTG has been used extensively to induce migraine-like hypersensitivity [17,18,19,20,43]. Our results are consistent with these previous studies: 10 mg/kg of NTG induces acute periorbital hypersensitivity, while 1 mg/kg of NTG is not enough to induce significant hypersensitivity. By combining MMTL with NTG injection, we mimicked the comorbid condition in a mouse model. MMTL pain is displayed in the masseter muscle (innervated by trigeminal nerve V3 branch) but not present in the periorbital area (innervated by trigeminal nerve V1 branch). NTG-induced acute migraine-like hypersensitivity was observed in the periorbital area innervated by trigeminal nerve V1. Interestingly, we further observed that MMTL pretreatment significantly prolonged NTG-induced facial mechanical allodynia in the periorbital area. Our results suggest that a pre-existing myogenic TMD can enhance NTG-induced migraine-like hypersensitivity and promote its chronicity. The combinative approach causes an exacerbated orofacial pain state. Moreover, a low dose (1 mg/kg) of NTG cannot induce detectable mechanical allodynia and light-aversive behavior as described previously [17], but MMTL pretreatment induces mechanical allodynia after injecting the low dose of NTG. Together, these results demonstrate that the new mouse model we developed can be used to mimic the comorbid migraine headache in TMD patients. However, this model has several limitations: (1) The MMTL we used produces myogenetic TMD, but TMDs in clinic are usually caused by a combination of factors; (2) Although NTG has been commonly used as a migraine triggering agent, the administration of NTG is systemic rather than restricted to specific trigeminal region; (3) In previous studies [20,44,45,46] light aversive behavior induced by NTG in rodents is observed only within 2 h, and we did not detect light aversive behavior 90 min or 24 h post-NTG administration. The timepoints of light-aversive behavior in rodents do not exactly match with the timeline of photophobia in patients with migraine. Therefore, further studies will be needed to determine the translational significance of our model. In the future, we may use other methods (such as genetic mouse model of migraine) to verify the effect of the MMTL-produced myogenic TMD on migraine pain.

To further validate that the periorbital facial pain enabled by MMTL pretreatment under low-dose of NTG is migraine-like, we used sumatriptan, an FDA-approved drug for migraine, in our studies. Our results showed that systemic injection of sumatriptan prior to NTG inhibits the MMTL-enabled periorbital hypersensitivity, indicating that the MMTL-enabled periorbital pain is migraine-like. Nitric oxide donors, such as NTG, can cause arterial dilatation and may induce migraine attack [47,48]. Sumatriptan is a potent selective serotonin receptor agonist that is used to abort migraine attacks [49,50], including NTG-induced migraine [17,51], by suppressing nitric oxide signaling [52,53]. However, it has been reported that the anti-migraine effect of sumatriptan involves both peripheral and central mechanisms [54]. This drug can regulate multiple signaling pathways by activating different types of serotonin receptors. Thus, sumatriptan may inhibit the MMTL-enhanced migraine-like hypersensitivity by modulating both nitric oxide-related and nitric oxide-irresponsive signaling pathways.

CGRP is synthesized in certain neurons and then released from their peripheral or central terminals in the trigeminal nociceptive system [55,56]. CGRP increases in the Sp5C in different migraine animal models [21,22,23]. In the present study, our results showed that the MMTL itself only increased the protein level of CGRP in the ipsilateral rostral Sp5C-V3 area, but not the caudal Sp5C-V1 area. However, when combined with NTG, the MMTL significantly increased NTG-induced CGRP in the caudal Sp5C-V1 at different time points. These results suggest that CGRP in the Sp5C not only contributes to the molecular mechanisms for TMDs and migraine, but also plays a role in their comorbidity. The released higher CGRP after MMTL could activate trigeminal nociceptive neurons in the Sp5C and facilitate central sensitization, thereby promoting chronicity of migraine pain. A previous study [57] reported that CGRP expression in the Sp5C decreases at 4 h after subcutaneous injection of NTG. The disparity of NTG-produced change in CGRP protein level may be due to different tissue harvest time and the NTG injection method used in that study and our study.

Previous studies have shown that both inflammation in the TMJ [58,59] and inflammation on the dura [60] produce inflammatory response in the trigeminal ganglion. Trigeminal ganglion inflammation might play a role in the pathogenesis of migraine [61]. The inflammation in one of the ganglion branches could lead to increased likelihood of hypersensitivity in other branches, which may contribute to the development of migraine and its chronicity. In the future, we will target trigeminal ganglion to investigate how the ganglion inflammation affects migraine and produces exacerbated orofacial pain in our combination model. 

In conclusion, we combined a myogenic TMD with NTG-induced migraine-like hypersensitivity to mimic the comorbidity of TMD and migraine in a mouse model. Our results indicate that a pre-existing myogenic TMD enhances NTG-induced migraine-like hypersensitivity, which could be mediated by upregulation of CGRP in the Sp5C. In the future, we will use this mouse model to investigate the underlying mechanisms of this comorbidity. It is hoped that our work will help identify potential therapeutic targets for such comorbid condition in order to develop a mechanism-based, novel therapy for patients with these frequently occurring pain presentations.

## 4. Materials and Methods

### 4.1. Animals

Totally 204 male C57BL/6 mice (6–8 weeks, Charles River Laboratories, Wilmington, MA, USA) were used. The mice were housed under standard conditions on a 14:10 light/dark cycle (7 AM–9 PM light), with food and water available ad libitum. The mice were randomly assigned into different groups. All behavioral tests were carried out by an investigator blinded to the treatment groups. All experiments were approved by the Texas A&M University Institutional Animal Care and Use Committee and all animal procedures were carried out in accordance with the National Institutes of Health Guide for the Care and Use of Laboratory Animals and the Animal Research Reporting of In Vivo Experiments (ARRIVE) guidelines.

### 4.2. MMTL Surgery

Unilateral MMTL was conducted with modification from a rat model [15]. Briefly, mice were anesthetized with intraperitoneal (i.p.) injection of pentobarbital sodium (50 mg/kg). MMTL was achieved via an intraoral approach. On the left side, the tendon of the anterior superficial part of the masseter muscle was gently freed from the surrounding connective tissues and then tied with two 6.0 chromic gut ligatures. The ligatures are 2 mm apart. The incision was closed with Vetbond tissue adhesive (Catalog #1469SB, 3M, St. Paul, MN, USA). The sham control mice underwent the same operation, but the tendon was not ligated. 

### 4.3. Drug Administration

A stock solution of NTG (5.0 mg/mL, NDC #0517-4810-25, American Regent, Shirley, NY, USA) containing 30% alcohol and 30% propylene glycol was freshly diluted with 0.9% saline. The vehicle control for NTG was its dissolvent (30% alcohol and 30% propylene glycol) diluted in 0.9% saline. Mice received a single injection of NTG or vehicle (i.p.), and NTG included two doses: 10 mg/kg (the higher dose) and 1 mg/kg (the lower dose). To validate MMTL-produced myogenic TMD pain, ibuprofen (Catalog # I1892, Sigma-Aldrich, St. Louis, MO, USA) was dissolved in 0.9% saline and then administered by oral gavage at two doses (30 mg/kg and 100 mg/kg) as described in recent publications [62,63,64,65,66]. For sumatriptan administration (one dosage), 20 min prior to NTG injections, each animal was injected (i.p.) with sumatriptan succinate (1 mg/kg in 0.9% saline, Catalog # S1198, Sigma-Aldrich, St. Louis, MO, USA) or 0.9% saline as described previously [17,67,68,69,70,71,72].

### 4.4. Functional Allodynia Test

TMD-produced functional allodynia was measured using a dolognawmeter, a validated operant assay to quantify gnawing dysfunction in mice [41]. Briefly, each mouse was placed in a tube confinement with one end closed. On the other end, two polymer dowels in series blocked mouse from escaping. The mice instinctually gnaw through both dowels to escape. The duration of time required to sever the second dowel was recorded as gnaw time. We started all tests at ~10:00 AM and trained all mice for 10 sessions before the MMTL or sham surgery. For each mouse, the average gnaw time of the last three training sessions served as its own baseline. Gnawing function was reported as a percent change from baseline.

### 4.5. Facial Mechanical Allodynia Test

The facial mechanical allodynia of mice was measured with calibrated von Frey filaments as described in our previous studies [20,36]. Briefly, each mouse was placed in an acrylic restraining cylinder (10 cm long, 3 cm internal diameter) and allowed to poke out its head and forepaws but cannot turn around [18]. The mice were habituated for 10 min prior to every test. A series of von Frey filaments (0.07, 0.16, 0.4, 0.6, 1.0, 1.4, and 2.0 g) were applied to the periorbital region ipsilateral to MMTL surgery (innervated by trigeminal nerve V1 branch) [73,74] and the skin area over the masseter muscle (innervated by trigeminal nerve V3 branch). Each filament, starting from the lowest force (0.07g) continuing in ascending order, was applied five times and each time lasted for 1–2 s with a 10 s interval. A positive response was defined as a clear withdrawal of the head or a forepaw swipe. The head withdrawal threshold was calculated as the force at which the positive response occurred at least three times out of five stimuli. 

### 4.6. Light-Aversive Behavior Test

Light-aversive behavior was examined via the light/dark box test on day 8 after MMTL (before NTG administration), 90 min and 24 h after NTG administration. The light/dark box test was carried out as described in a previous study [20]. Briefly, a light/dark box was custom made (30 × 30 × 30 cm) with two equal-sized compartments and a small opening (7 × 7 cm) connecting the two compartments. The light compartment was painted white without a cover and a LED illuminator (1000 lx, Catalog #AMPS-ILED-21, Laxco Inc, Mill Creek, WA, USA) was placed over the top; the dark compartment was painted black with covered top. Mice were individually tested in the custom-made light/dark box for 30 min. At the beginning of the test, mice were put at the small opening door between the two compartments, so that the mice can choose which compartment they prefer to enter. The box was cleaned with 75% ethanol followed by distilled water between each test. The test sessions were recorded using a video camera, without the presence of the experimenter in the test room; this was done to minimize potential environmental variables introduced by the experimenter. The videos were evaluated later and the percentage of time spent in the dark compartment was calculated.

### 4.7. Hematoxylin and Eosin (H&E) Staining

On days 3, 7 and 15 after MMTL surgery, mice were perfused intracardially with 4% paraformaldehyde (PFA) in 0.1 M PBS (pH 7.4) under deep anesthesia with isoflurane. The masseter muscle and tendon tissues were harvested, post-fixed in 4% PFA in 0.1 M PBS for 24 h, and cryoprotected in 30% (w/v) sucrose for 48 h at 4 °C. The tissues were sectioned at 15 μm thickness with a cryostat (CM1950, Leica, Buffalo Grove, IL, USA). Standard H&E staining was performed according to the manufacturer′s instructions (Catalog #MHS16 and #HT110116, Sigma-Aldrich, USA). 

### 4.8. Immunohistochemistry

On day 7 after MMTL surgery, and at 2 or 24 h after NTG administration, mice were perfused as described above. Next, the brainstem tissues containing Sp5C were harvested, post-fixed, and cryoprotected. The tissues were sectioned at 20 μm thickness. For each mouse, sections were collected (one section from every five consecutive sections) and blocked in 0.1 M PBS containing 5% normal goat serum (Catalog #5425S, Cell Signaling, Danvers, MA, USA) and 0.3% Triton X-100 for 1 h. The sections were incubated with mouse anti-calcitonin gene-related peptide (CGRP) antibody (1:300; Catalog # ab81887, Abcam, Cambridge, MA, USA) diluted in blocking solution for 20 h at 4 °C, and then rinsed and incubated with a Cy3 conjugated secondary antibody (1:400; Catalog # 715-165-150, Jackson ImmunoResearch, West Grove, PA, USA) for 1 h at room temperature. The sections were examined under a Leica microscope (DMi8, Leica, Buffalo Grove, IL, USA). For quantification of immunohistochemical staining, images of caudal Sp5C-V1 and rostral Sp5C-V3 [75] were taken under the 40X objective lens, at 500-ms exposure time. Total integrated density of all the positive signals was measured using Leica Application Suite X (LAS X software, Leica, Buffalo Grove, IL, USA). For each mouse, three sections were used.

### 4.9. Statistical Analysis

All data are expressed as means ± standard error of mean (SEM). Statistical analyses were performed with SPSS Statistics, version 24.0 (IBM Corp, Armonk, NY, USA). Sample size calculations were performed using the power analysis program G*Power 3.1 [76]. The data from behavioral tests were analyzed with two-way repeated measures ANOVA followed by Sidak or Tukey’s post hoc test. The immunohistochemistry data were analyzed with two-way ANOVA followed by Tukey’s post hoc test. Statistical significance was accepted for a *p*-value less than 0.05. 

## Supplementary Materials

The following are available online at https://www.mdpi.com/1422-0067/21/11/4049/s1; **Supplementary Figure S1**: Systemic injection of NTG induces mechanical hypersensitivity in trigeminal nerve V3-innervated masseter area of mice; **Supplementary Figure S2**: MMTL differentially affects NTG-induced mechanical hypersensitivity in ipsilateral and contralateral periorbital area; **Supplementary Figure S3**: MMTL enhances NTG-induced migraine-like hypersensitivity in female mice; **Supplementary Figure S4**: Specificity of the CGRP antibody. Sp5C-containing brainstem tissues from wild-type mice were harvested for immunofluorescence staining.

## Abbreviation List

CGRP: calcitonin gene-related peptide
MMTL: masseter muscle tendon ligation
NTG: nitroglycerin
Sp5C: spinal trigeminal nucleus caudalis
TMD: temporomandibular disorders
TMJ: temporomandibular joint

## References

[1] Burch R.C., Loder S., Loder E., Smitherman T.A. (2015). The prevalence and burden of migraine and severe headache in the United States: Updated statistics from government health surveillance studies. Headache.

[2] Schiffman E., Ohrbach R., Truelove E., Look J., Anderson G., Goulet J.P., List T., Svensson P., Gonzalez Y., Lobbezoo F. (2014). Diagnostic Criteria for Temporomandibular Disorders (DC/TMD) for Clinical and Research Applications: Recommendations of the International RDC/TMD Consortium Network* and Orofacial Pain Special Interest Groupdagger. J. Oral Facial Pain Headache.

[3] Maixner W., Diatchenko L., Dubner R., Fillingim R.B., Greenspan J.D., Knott C., Ohrbach R., Weir B., Slade G.D. (2011). Orofacial Pain Prospective Evaluation and Risk Assessment Study—The OPPERA Study. J. Pain.

[4] National Institute of Dental and Craniofacial Research Prevalence of TMJD and Its Signs and Symptoms. https://www.nidcr.nih.gov/research/data-statistics/facial-pain/prevalence.

[5] Tjakkes G.H., Reinders J.J., Tenvergert E.M., Stegenga B. (2010). TMD pain: The effect on health related quality of life and the influence of pain duration. Health Qual. Life Outcomes.

[6] Feigin V.L., Abajobir A.A., Abate K.H., Abd-Allah F., Abdulle A.M., Abera S.F., Abyu G.Y., Ahmed M.B., Aichour A.N., Aichour I. (2017). Global, regional, and national burden of neurological disorders during 1990–2015: A systematic analysis for the Global Burden of Disease Study 2015. Lancet Neurol..

[7] Vos T., Barber R.M., Bell B., Bertozzi-Villa A., Biryukov S., Bolliger I., Charlson F., Davis A., Degenhardt L., Dicker D. (2015). Global Burden of Disease Study C. Global, regional, and national incidence, prevalence, and years lived with disability for 301 acute and chronic diseases and injuries in 188 countries, 1990-2013: A systematic analysis for the Global Burden of Disease Study 2013. Lancet.

[8] Ballegaard V., Thede-Schmidt-Hansen P., Svensson P., Jensen R. (2008). Are headache and temporomandibular disorders related? A blinded study. Cephalalgia.

[9] Mitrirattanakul S., Merrill R.L. (2006). Headache impact in patients with orofacial pain. J. Am. Dent. Assoc..

[10] Tchivileva I.E., Ohrbach R., Fillingim R.B., Greenspan J.D., Maixner W., Slade G.D. (2017). Temporal change in headache and its contribution to the risk of developing first-onset temporomandibular disorder in the Orofacial Pain. Pain.

[11] Goncalves D.A., Bigal M.E., Jales L.C., Camparis C.M., Speciali J.G. (2010). Headache and symptoms of temporomandibular disorder: An epidemiological study. Headache.

[12] Dahan H., Shir Y., Velly A., Allison P. (2015). Specific and number of comorbidities are associated with increased levels of temporomandibular pain intensity and duration. J. Headache Pain.

[13] Contreras E.F.R., Fernandes G., Ongaro P.C.J., Campi L.B., Goncalves D.A.G. (2018). Systemic diseases and other painful conditions in patients with temporomandibular disorders and migraine. Braz. Oral Res..

[14] Speciali J.G., Dach F. (2015). Temporomandibular dysfunction and headache disorder. Headache.

[15] Guo W., Wang H., Zou S., Wei F., Dubner R., Ren K. (2010). Long lasting pain hypersensitivity following ligation of the tendon of the masseter muscle in rats: A model of myogenic orofacial pain. Mol. Pain.

[16] Kramer P.R., Bellinger L.L. (2014). Infusion of Gabralpha6 siRNA into the trigeminal ganglia increased the myogenic orofacial nociceptive response of ovariectomized rats treated with 17beta-estradiol. Neuroscience.

[17] Bates E.A., Nikai T., Brennan K.C., Fu Y.H., Charles A.C., Basbaum A.I., Ptacek L.J., Ahn A.H. (2010). Sumatriptan alleviates nitroglycerin-induced mechanical and thermal allodynia in mice. Cephalalgia.

[18] Farkas S., Bolcskei K., Markovics A., Varga A., Kis-Varga A., Kormos V., Gaszner B., Horvath C., Tuka B., Tajti J. (2016). Utility of different outcome measures for the nitroglycerin model of migraine in mice. J. Pharmacol. Toxicol. Methods.

[19] Markovics A., Kormos V., Gaszner B., Lashgarara A., Szoke E., Sandor K., Szabadfi K., Tuka B., Tajti J., Szolcsanyi J. (2012). Pituitary adenylate cyclase-activating polypeptide plays a key role in nitroglycerol-induced trigeminovascular activation in mice. Neurobiol. Dis..

[20] Tang Y., Liu S., Shu H., Xing Y., Tao F. (2018). AMPA receptor GluA1 Ser831 phosphorylation is critical for nitroglycerin-induced migraine-like pain. Neuropharmacology.

[21] Dong X., Hu Y., Jing L., Chen J. (2015). Role of phosphorylated extracellular signal-regulated kinase, calcitonin gene-related peptide and cyclooxygenase-2 in experimental rat models of migraine. Mol. Med. Rep..

[22] Greco R., Demartini C., Zanaboni A.M., Tassorelli C. (2018). Chronic and intermittent administration of systemic nitroglycerin in the rat induces an increase in the gene expression of CGRP in central areas: Potential contribution to pain processing. J. Headache Pain.

[23] Yao G., Han X., Hao T., Huang Q., Yu T. (2015). Effects of rizatriptan on the expression of calcitonin gene-related peptide and cholecystokinin in the periaqueductal gray of a rat migraine model. Neurosci. Lett..

[24] Dym H., Israel H. (2012). Diagnosis and treatment of temporomandibular disorders. Dent. Clin. N. Am..

[25] Gauer R.L., Semidey M.J. (2015). Diagnosis and treatment of temporomandibular disorders. Am. Fam. Physician.

[26] Ouanounou A., Goldberg M., Haas D.A. (2017). Pharmacotherapy in Temporomandibular Disorders: A Review. J. Can. Dent. Assoc..

[27] Hersh E.V., Balasubramaniam R., Pinto A. (2008). Pharmacologic management of temporomandibular disorders. Oral Maxillofac. Surg. Clin. N. Am..

[28] Ho T.W., Edvinsson L., Goadsby P.J. (2010). CGRP and its receptors provide new insights into migraine pathophysiology. Nat. Rev. Neurol..

[29] Bender S.D. (2014). Orofacial Pain and Headache: A Review and Look at the Commonalities. Curr. Pain Headache Rep..

[30] Dando W.E., Branch M.A., Maye J.P. (2006). Headache disability in orofacial pain patients. Headache.

[31] Stuginski-Barbosa J., Macedo H.R., Bigal M.E., Speciali J.G. (2010). Signs of temporomandibular disorders in migraine patients: A prospective, controlled study. Clin. J. Pain.

[32] Suzuki A., Iwata J. (2016). Mouse genetic models for temporomandibular joint development and disorders. Oral Dis..

[33] Kumagai K., Suzuki S., Kanri Y., Matsubara R., Fujii K., Wake M., Suzuki R., Hamada Y. (2015). Spontaneously developed osteoarthritis in the temporomandibular joint in STR/ort mice. Biomed. Rep..

[34] Xu L., Polur I., Lim C., Servais J.M., Dobeck J., Li Y., Olsen B.R. (2009). Early-onset osteoarthritis of mouse temporomandibular joint induced by partial discectomy. Osteoarthr. Cartil..

[35] Romero-Reyes M., Pardi V., Akerman S. (2015). A potent and selective calcitonin gene-related peptide (CGRP) receptor antagonist, MK-8825, inhibits responses to nociceptive trigeminal activation: Role of CGRP in orofacial pain. Exp. Neurol..

[36] Bai Q., Liu S., Shu H., Tang Y., George S., Dong T., Schmidt B.L., Tao F. (2019). TNFalpha in the Trigeminal Nociceptive System Is Critical for Temporomandibular Joint Pain. Mol. Neurobiol..

[37] Widmer C.G., Morris-Wiman J. (2015). Assessment of incising ethology in the absence and presence of jaw muscle hyperalgesia in a mouse home cage environment. Physiol. Behav..

[38] Wong H., Kang I., Dong X.D., Christidis N., Ernberg M., Svensson P., Cairns B.E. (2014). NGF-induced mechanical sensitization of the masseter muscle is mediated through peripheral NMDA receptors. Neuroscience.

[39] Hansdottir R., Bakke M. (2004). Joint tenderness, jaw opening, chewing velocity, and bite force in patients with temporomandibular joint pain and matched healthy control subjects. J. Orofac. Pain.

[40] Bakke M., Hansdottir R. (2008). Mandibular function in patients with temporomandibular joint pain: A 3-year follow-up. Oral Surg. Oral Med. Oral Pathol. Oral Radiol. Endod..

[41] Dolan J.C., Lam D.K., Achdjian S.H., Schmidt B.L. (2010). The dolognawmeter: A novel instrument and assay to quantify nociception in rodent models of orofacial pain. J. Neurosci. Methods.

[42] Ackermann P.W., Franklin S.L., Dean B.J., Carr A.J., Salo P.T., Hart D.A. (2014). Neuronal pathways in tendon healing and tendinopathy—Update. Front Biosci..

[43] Tang Y., Liu S., Shu H., Yanagisawa L., Tao F. (2020). Gut Microbiota Dysbiosis Enhances Migraine-Like Pain Via TNFalpha Upregulation. Mol. Neurobiol..

[44] Farajdokht F., Babri S., Karimi P., Mohaddes G. (2016). Ghrelin attenuates hyperalgesia and light aversion-induced by nitroglycerin in male rats. Neurosci. Lett..

[45] Harris H.M., Carpenter J.M., Black J.R., Smitherman T.A., Sufka K.J. (2017). The effects of repeated nitroglycerin administrations in rats; modeling migraine-related endpoints and chronification. J. Neurosci. Methods.

[46] Sufka K.J., Staszko S.M., Johnson A.P., Davis M.E., Davis R.E., Smitherman T.A. (2016). Clinically relevant behavioral endpoints in a recurrent nitroglycerin migraine model in rats. J. Headache Pain.

[47] Thomsen L.L., Kruuse C., Iversen H.K., Olesen J. (1994). A nitric oxide donor (nitroglycerin) triggers genuine migraine attacks. Eur. J. Neurol..

[48] Neeb L., Reuter U. (2007). Nitric oxide in migraine. CNS Neurol. Disord. Drug Targets.

[49] Silberstein S.D., Marcus D.A. (2013). Sumatriptan: Treatment across the full spectrum of migraine. Expert Opin. Pharmacother..

[50] Napoletano F., Lionetto L., Martelletti P. (2014). Sumatriptan in clinical practice: Effectiveness in migraine and the problem of psychiatric comorbidity. Expert Opin. Pharmacother..

[51] Fullerton T., Komorowski-Swiatek D., Forrest A., Gengo F.M. (1999). The pharmacodynamics of sumatriptan in nitroglycerin-induced headache. J. Clin. Pharmacol..

[52] Ikeda Y., Jimbo H., Shimazu M., Satoh K. (2002). Sumatriptan scavenges superoxide, hydroxyl, and nitric oxide radicals: In vitro electron spin resonance study. Headache.

[53] Read S.J., Manning P., McNeil C.J., Hunter A.J., Parsons A.A. (1999). Effects of sumatriptan on nitric oxide and superoxide balance during glyceryl trinitrate infusion in the rat. Implications for antimigraine mechanisms. Brain Res..

[54] Ahn A.H., Basbaum A.I. (2005). Where do triptans act in the treatment of migraine?. Pain.

[55] Amara S.G., Arriza J.L., Leff S.E., Swanson L.W., Evans R.M., Rosenfeld M.G. (1985). Expression in brain of a messenger RNA encoding a novel neuropeptide homologous to calcitonin gene-related peptide. Science.

[56] Iyengar S., Johnson K.W., Ossipov M.H., Aurora S.K. (2019). CGRP and the Trigeminal System in Migraine. Headache.

[57] Pardutz A., Multon S., Malgrange B., Parducz A., Vecsei L., Schoenen J. (2002). Effect of systemic nitroglycerin on CGRP and 5-HT afferents to rat caudal spinal trigeminal nucleus and its modulation by estrogen. Eur. J. Neurosci..

[58] Csati A., Edvinsson L., Vecsei L., Toldi J., Fulop F., Tajti J., Warfvinge K. (2015). Kynurenic acid modulates experimentally induced inflammation in the trigeminal ganglion. J. Headache Pain.

[59] Spears R., Dees L.A., Sapozhnikov M., Bellinger L.L., Hutchins B. (2005). Temporal changes in inflammatory mediator concentrations in an adjuvant model of temporomandibular joint inflammation. J. Orofac. Pain.

[60] Lukacs M., Haanes K.A., Majlath Z., Tajti J., Vecsei L., Warfvinge K., Edvinsson L. (2015). Dural administration of inflammatory soup or Complete Freund’s Adjuvant induces activation and inflammatory response in the rat trigeminal ganglion. J. Headache Pain.

[61] Edvinsson L., Haanes K.A., Warfvinge K. (2019). Does inflammation have a role in migraine?. Nat. Rev. Neurol..

[62] Alagan A., Jantan I., Kumolosasi E., Ogawa S., Abdullah M.A., Azmi N. (2019). Protective Effects of Phyllanthus amarus Against Lipopolysaccharide-Induced Neuroinflammation and Cognitive Impairment in Rats. Front Pharmacol..

[63] Bastaki S.M.A., Padol I.T., Amir N., Hunt R.H. (2018). Effect of Aspirin and ibuprofen either alone or in combination on gastric mucosa and bleeding time and on serum prostaglandin E2 and thromboxane A2 levels in the anaesthetized rats in vivo. Mol. Cell. Biochem..

[64] Giannakis N., Sansbury B.E., Patsalos A., Hays T.T., Riley C.O., Han X., Spite M., Nagy L. (2019). Dynamic changes to lipid mediators support transitions among macrophage subtypes during muscle regeneration. Nat. Immunol..

[65] Montilla-Garcia A., Tejada M.A., Perazzoli G., Entrena J.M., Portillo-Salido E., Fernandez-Segura E., Canizares F.J., Cobos E.J. (2017). Grip strength in mice with joint inflammation: A rheumatology function test sensitive to pain and analgesia. Neuropharmacology.

[66] Salama R.A.M., El Gayar N.H., Georgy S.S., Hamza M. (2016). Equivalent intraperitoneal doses of ibuprofen supplemented in drinking water or in diet: A behavioral and biochemical assay using antinociceptive and thromboxane inhibitory dose-response curves in mice. Peer J..

[67] Huang D., Ren L., Qiu C.S., Liu P., Peterson J., Yanagawa Y., Cao Y.Q. (2016). Characterization of a mouse model of headache. Pain.

[68] Mitsikostas D.D., Sanchez del Rio M., Waeber C. (2002). 5-Hydroxytryptamine(1B/1D) and 5-hydroxytryptamine1F receptors inhibit capsaicin-induced c-fos immunoreactivity within mouse trigeminal nucleus caudalis. Cephalalgia.

[69] Ferrari L.F., Levine J.D., Green P.G. (2016). Mechanisms mediating nitroglycerin-induced delayed-onset hyperalgesia in the rat. Neuroscience.

[70] Khalilzadeh M., Panahi G., Rashidian A., Hadian M.R., Abdollahi A., Afshari K., Shakiba S., Norouzi-Javidan A., Rahimi N., Momeny M. (2018). The protective effects of sumatriptan on vincristine—Induced peripheral neuropathy in a rat model. Neurotoxicology.

[71] Nation K.M., Dodick D.W., Navratilova E., Porreca F. (2019). Sustained exposure to acute migraine medications combined with repeated noxious stimulation dysregulates descending pain modulatory circuits: Relevance to medication overuse headache. Cephalalgia.

[72] Sandweiss A.J., Cottier K.E., McIntosh M.I., Dussor G., Davis T.P., Vanderah T.W., Largent-Milnes T.M. (2017). 17-beta-Estradiol induces spreading depression and pain behavior in alert female rats. Oncotarget..

[73] Elliott M.B., Oshinsky M.L., Amenta P.S., Awe O.O., Jallo J.I. (2012). Nociceptive neuropeptide increases and periorbital allodynia in a model of traumatic brain injury. Headache.

[74] Romero-Reyes M., Ye Y. (2013). Pearls and pitfalls in experimental in vivo models of headache: Conscious behavioral research. Cephalalgia.

[75] Abdallah K., Artola A., Monconduit L., Dallel R., Luccarini P. (2013). Bilateral descending hypothalamic projections to the spinal trigeminal nucleus caudalis in rats. PLoS ONE.

[76] Faul F., Erdfelder E., Lang A.G., Buchner A. (2007). G*Power 3: A flexible statistical power analysis program for the social, behavioral, and biomedical sciences. Behav. Res. Methods.

