# Supplementary material for: A Pre-Existing Myogenic Temporomandibular Disorder Increases Trigeminal Calcitonin Gene-Related Peptide and Enhances Nitroglycerin-Induced Hypersensitivity in Mice
Source: Int J Mol Sci. 2020 Jun 5;21(11):4049. doi: 10.3390/ijms21114049 (PMC7313473; doi:10.3390/ijms21114049)
Supplement: Supplementary file 1 [file ijms-21-04049-s001.pdf]

International Journal of
Molecular Sciences

MDPI

Supplementary Materials

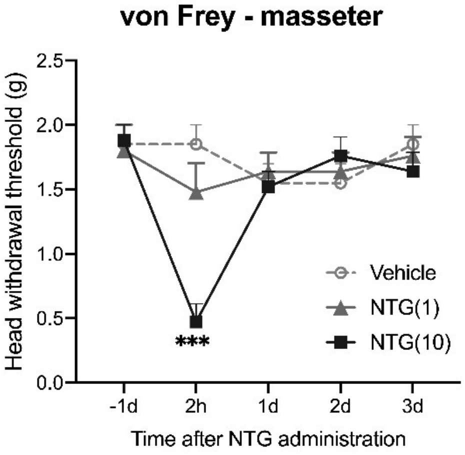

Supplemental Figure 1. Systemic injection of NTG induces mechanical hypersensitivity in trigeminal nerve V3-innervated masseter area of mice. Intraperitoneal injection (i.p.) of NTG at 10 mg/kg, but not 1 mg/kg, decreased the head withdrawal threshold in the skin area over masseter muscle (n = 5 per group). ***$P < 0.001$ *vs* the vehicle-treated group. NTG, nitroglycerin; NTG(1): 1 mg/kg of nitroglycerin; NTG(10): 10 mg/kg of nitroglycerin.

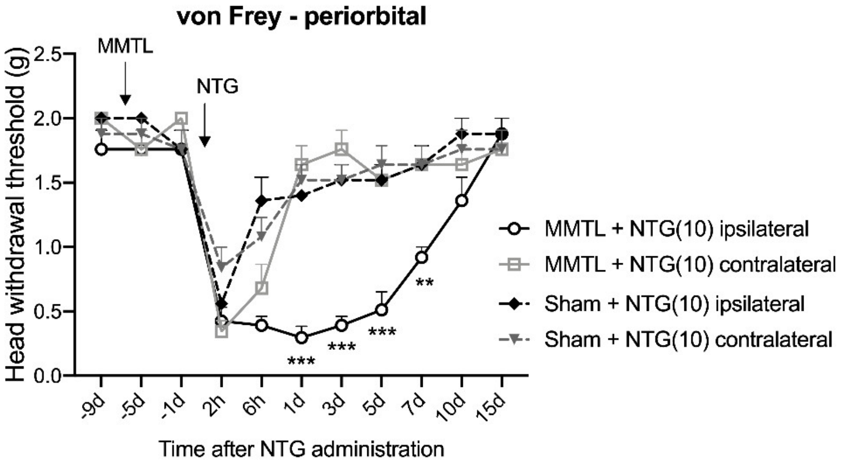

Supplemental Figure 2. MMTL differentially affects NTG-induced mechanical hypersensitivity in ipsilateral and contralateral periorbital area. We observed that the MMTL significantly extended the NTG-decreased head withdrawal threshold in the ipsilateral periorbital area, but only slightly prolonged the NTG-decreased head withdrawal threshold on the contralateral side (n = 5 per group). **$P < 0.01$, ***$P < 0.001$ *vs* the respective " MMTL + NTG(10) contralateral" group. $F_{treatment}$ (3, 16) = 29.950, $P < 0.001$; F_{time} (10, 160) = 51.510, $P < 0.001$; $F_{interaction}$ (30, 160) = 4.914, $P < 0.001$. NTG(10), 10 mg/kg of nitroglycerin.

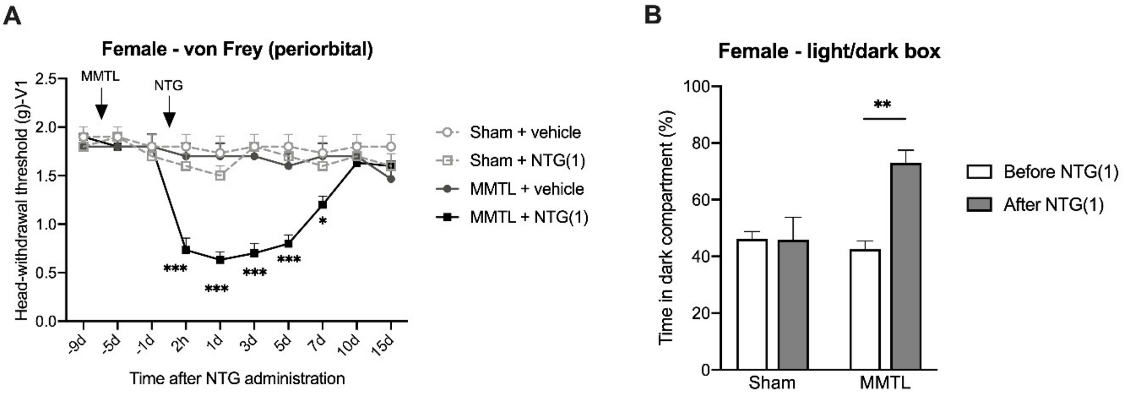

Supplemental Figure 3. MMTL enhances NTG-induced migraine-like hypersensitivity in female mice. **(A)** MMTL pretreatment enabled the lower dose (1 mg/kg) of NTG to decrease the head withdrawal threshold in the periorbital region (n = 6 per group). $F_{treatment}$ (3, 20) = 8.700, $P < 0.001$; F_{time} (9, 180) = 9.98, $P < 0.001$; $F_{interaction}$ (27, 180) = 5.46, $P < 0.001$. ***$P < 0.001$, *$P < 0.05$ *vs* the corresponding time points in the "sham + vehicle" group. **(B)** MMTL pretreatment enabled the lower dose (1 mg/kg) of NTG to produce the light-aversive behavior (n = 6 per group). $F_{treatment}$ (1, 10) = 6.879, $P < 0.05$; F_{time} (1, 10) = 7.520, $P < 0.05$; $F_{interaction}$ (1, 10) = 7.798, $P < 0.05$. **$P < 0.01$ as indicated in the figure. MMTL: masseter muscle tendon ligation; NTG: nitroglycerin; NTG(1): 1 mg/kg of nitroglycerin.

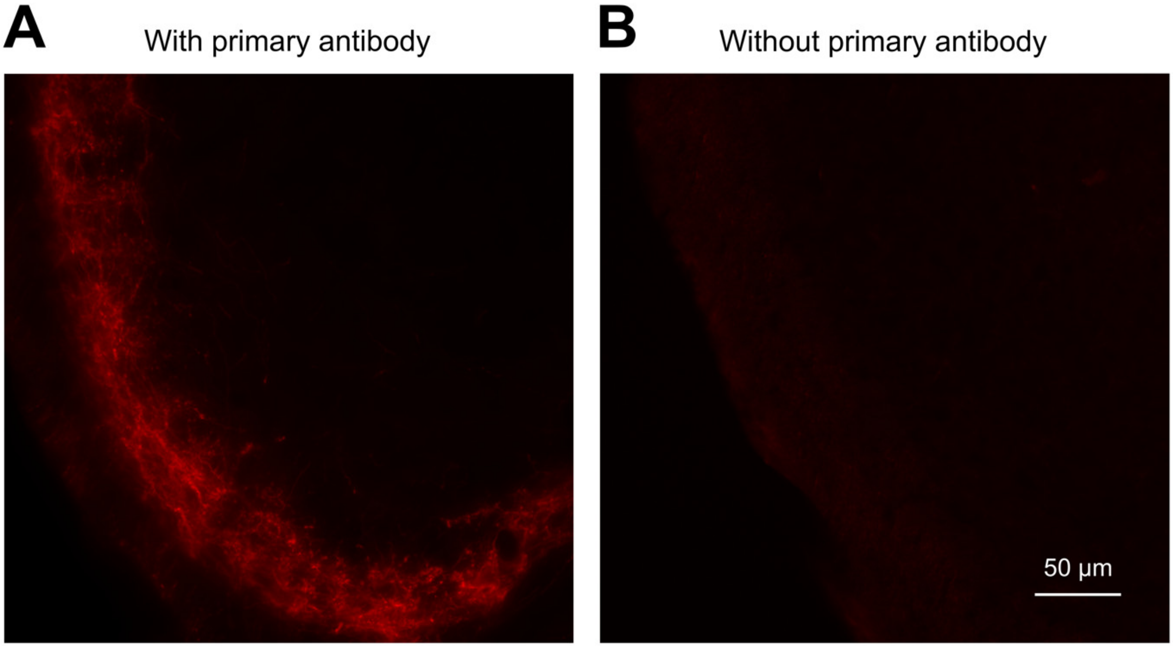

Supplemental Figure 4. Specificity of the CGRP antibody. Sp5C-containing brainstem tissues from wild-type mice were harvested for immunofluorescence staining. (**A**) The staining with primary CGRP antibody showed CGRP-positive signals in the superficial laminae of the Sp5C. (**B**) The staining with the primary CGRP antibody omitted showed no positive signals in the area.
